# Long non-coding RNA steroid receptor activator promotes the progression of endometrial cancer via Wnt/ β-catenin signaling pathway

**DOI:** 10.7150/ijbs.35643

**Published:** 2020-01-01

**Authors:** Sun-Ae Park, Lee Kyung Kim, Young Tae Kim, Tae-Hwe Heo, Hee Jung Kim

**Affiliations:** 1 Laboratory of Pharmacoimmunology, Integrated Research Institute of Pharmaceutical Sciences, College of Pharmacy, The Catholic University of Korea, Seoul, 03722, Republic of Korea; 2 Institute of Women's Life Medical Science, Division of Gynecologic Oncology, Department of Obstetrics and Gynecology, Yonsei University College of Medicine, Seoul, 03722, Republic of Korea

**Keywords:** Endometrial cancer (EC), Steroid Receptor Activator (*SRA*), Signaling pathway, Eukaryotic translation initiation factor 4E-binding protein 1 (EIF4E-BP1), Wnt/ β-catenin signaling

## Abstract

**Rationale:** Steroid receptor activator (*SRA*), a long non-coding RNA, serves as a critical regulator of gynecologic cancer. The objective of this study was to determine biological function and clinical significance of *SRA* expression in endometrial cancer.

**Method:** We investigated whether *SRA* was involved in the development of endometrial cancer via binding to eukaryotic translation initiation factor 4E-binding protein 1 (EIF4E-BP1) as a transcription factor to enhance Wnt/ β-catenin signaling pathway.

**Results:** Expression levels of *SRA* were upregulated in endometrial cancer tissues compared to those in adjacent control tissues. We also found high expression of *SRA* in EC cells. The relationship between *SRA* and EIF4E-BP1 was corroborated by transfection of a luciferase reporter plasmid. In addition, *SRA* knockdown inhibited the expression of EIF4E-BP1 known to play a critical role in the control of protein synthesis, cell growth, and cell survival, thus promoting tumourigenesis and epithelial-mesenchymal transition (EMT) important for cell motility and metastasis. Consistently, immunostaining and western blotting analysis showed that expression levels of β-catenin and 4EBP1 in the nucleus were significantly decreased by *SRA* knockdown but increased by *SRA* over-expression.

**Conclusions:** These results suggest that *SRA* is involved in proliferation, migration, and invasion of endometrial cancer cells by increasing the expression of EIF4E-BP1 and activity of Wnt/ β-catenin signaling. These findings indicate that *SRA* might be a novel biomarker for predicting recurrence and prognosis. It might also serve as a promising therapeutic target in endometrial cancer.

## Introduction

Endometrial cancer (EC) is one of the most common gynecologic cancer. The incidence of EC has remarkable increased in recent years. EC rarely occurs in women before the age of 40 years or after the age of 70 years. Its prognosis is poor. The 5-year survival rate of endometritis with appropriate treatment is 80%. 1 The recurrence rate of uterine hysterectomy in early stage is 3~17% depending on whether primary treatment or adjuvant therapy is performed. Most cases of recurrence occur after 2 to 3 years of treatment (64% after 2 years and 87% after 3 years). 2 Thus, recent research has focused on finding tumor-specific markers that can predict the biological behavior of EC in relation to cellular motility and invasion. 3 However, pathological features and prognosis of patients with EC remain controversial. Recent studies have shown that non-coding RNA may be involved in the development of EC. 4, 5

Non-coding RNAs (ncRNAs) is a key component that can influence gene regulation and cancer cell phenotypes. 6, 7 Increasing number of studies have confirmed the presence of lncRNA in the cytoplasm and found that lncRNA is also involved in translation and post-translational regulation of gene expression. 8, 9 LncRNA transcription is highly regulated. 10, 11 In addition, lncRNA may contain various types of binding domains to allowing binding with effector and repressor molecules as well as binding of protein complexes to larger functional units. 7, 12 Recently, it has been shown that lncRNA plays a role in biological events such as cell growth and differentiation.

The steroid receptor RNA activator (*SRA*) was identified as functional RNA transcript existing in a ribonucleoprotein complex. That can co-activate steroid nuclear receptors. *SRA* was located on human chromosome 5q31.3 containing 5 exons and 4 introns. *SRA* was approximately 0.87 kB in size. *SRA* is both a protein-coding and non-coding RNA. 13-15 LncRNA *SRA* with an important function in tumor. It functions as a molecular coactivator for the expression of genes encoding *SRA* such as progesterone and estrogen in the development of cancer. It has been shown that *SRA* can activate hormone receptors that are associated with gynecologic cancers such as ovarian cancer and breast cancer. LncRNA *SRA* has been implicated in normal biological processes such as apoptosis, lipogenesis, steroidogenesis, muscle formation, and insulin signaling. It has also been shown to play a role in breast cancer, prostate cancer, abnormal cardiac development, and fertility reduction. 16, 17 In addition, lncRNA *SRA* has been studied in relation to tumor progression, although the mechanism is insufficient. To elucidate lncRNA SRA mechanism through EIF4E-PB1, which is known to be a downstream target for this cell growth and proliferation.

Eukaryotic translation initiation factor 4E-binding protein 1 (EIF4E-BP1) belongs to a family of translation-repressor proteins. It is one of two main downstream effectors of mammalian target of rapamycin (mTOR). 18, 19 EIF4E-BP1 is known to have important effect on mTOR signaling through translational control of key carcinogenic mRNA encoding proteins for cell cycle progression, cell survival, angiogenesis, cancer progression and metastasis. EIF4E-BP1 expression is regulated by transcriptional and post-translational mechanisms. 20-22 EIF4E-BP1 is an oncogene that is overexpressed in a wide range of cancers. 23

Accordingly, in this study, we investigated expression levels of *SRA* in EC patient tissues and analyzed the relationship among *SRA* expression, clinicopathological findings, and disease prognosis. Functional analysis was also performed to examine effects of *SRA* on invasion and migration of EC cells both *in vitro* and *in vivo*. Finally, we examined whether *SRA* was involved in the development of EC cells via EIF4E-BP1 mediated Wnt/ β-catenin regulation.

## Materials and Methods

### Tissue collection

All experiments were performed with approval from the review board for human research of Yonsei University Hospital. Tissue samples of endometrial patients were collected at the time of surgery. These samples were immediately snap-frozen in liquid nitrogen and kept at -80℃ until RNA extraction.

### Cell culture

ECC-1 and Ishikawa EC cell lines were purchased from Sigma-Aldrich and ATCC. These cell lines were maintained in Minimum Essential Medium (MEM; Welgene Inc., Daegu, Korea) and RPMI-1640 (Welgene Inc) at 37℃ in an atmosphere of 5% CO_2_. Culture medium was replaced with fresh medium every 2-3 days.

### Transfection of siRNAs

siRNAs (*SRA* and negative control (siNC)) were purchased from Genolution (Genolution Phamaceutical Inc, Seoul, Korea). Cells were added to 6-well plates at density of 5×10^4^ cells/well. To transfect these cells with 10 nM siRNA in phosphate-buffered saline (PBS), a G-Fectin Kit (Genolution Phamaceutical Inc) was used according to the manufacturer's instructions. At 48 h post transfection, siRNA transfected cells were used for *in vitro* assay. Target siRNA sequences were listed in Supplementary Table 1, 2.

### Plasmid constructs and generation of stable cell lines

PCR was used to amplify human *SRA* transcript variant 3 cDNA. It was then inserted into a pLenti6/V-5-TOPO vector (ViraPower™ Lentiviral Expression Systems, Invitrogen, Carlsbad, CA, USA). The plasmid was then transfected into 293FT cell line for packaging. The resultant lentivirus was used to infect cell lines. Medium containing blasticidin (Invitrogen, Karlsruhe, Germany) was used for selecting *SRA* stably transfected cells. Plasmid pLKO.1-puro (control TRC shRNA vector) was used to transfect ECC-1 and Ishikawa cells. Cells transfected with the pLKO.1-puro plasmid were selected with 4 μg/ml puromycin and stable clones were maintained with 1 μg/ml puromycin. Three stable clones were isolated. Transfection was performed using Lipofectamine 2000 (Invitrogen) following the manufacturer's instructions.

### Quantitative real-time PCR analysis (qPCR)

RNAs were extracted from samples and cell lines (TRIzol^®^ reagent, Invitrogen). For reverse-transcription of 2 µg total RNA into first-strand cDNA, a reverse transcription kit (Invitrogen) was used according to the manufacturer's instructions. qRT-PCR was performed using SYBR^®^ Green real-time PCR kit (Mbiotech, Seoul, Korea) in a 20 µl reaction volume on an ABI StepOnePlus Real-Time PCR system (Applied Biosystems, Foster City, CA, USA). β-actin was used as internal standard to normalize gene expression levels. The 2^-∆∆CT^ method was used to analyze relative gene expression. Each qRT-PCR experiment was replicated ≥ 3 times. Results are expressed as extent of change relative to control values. Primers used for PCR reactions are listed in Supplementary Table 3.

### Luciferase reporter assay

NCBI (http://www.ncbi.nlm.nih.gov) online database was used to predict potential transcription factor binding sites at EIF4E-BP1 promoter regions. Several SP1 binding motifs were identified. The EIF4E-BP1 promoter region (651 bp) was then synthesized and inserted into a pGL4-basic vector (Promega, Madison, WI, USA). Successful integration of this sequence into the vector was verified by sequencing. A Dual-Luciferase Assay Kit (Promega Inc) was used to assess luciferase activities following manufacturer's protocol.

### Western blot analysis

Cells were lysed in 500 µl RIPA buffer (150 mM sodium chloride, 1% NP 40, 0.5% sodium deoxycholate, 0.1% sodium dodecyl sulphate, 50 mM Tris-HCl [pH 8.0], 100 mM PMSF) and centrifuged at 14,000 g for 15 min at 4℃. The supernatant was mixed with denaturing sample buffer (1:1) and boiled at 95℃ for 5 min. Equal amounts of protein (30 μg) were loaded and separated by 10% SDS-polyacrylamide gel electrophoresis and blotted onto PDVF membranes (Sigma-Aldrich, St Louis, MO, USA). These membranes were blocked with 5% non-fat dry milk in Tris-buffered saline containing 0.05% Tween 20 (TBST) for 1 h at 4℃ and incubated with anti-β-catenin (1:1000), anti-VEGF (1:1000), anti-BCL-2 (1:500), anti-Bax (1:1000), anti-APAF-1 (1:1000), anti-Caspase-9 (1:500), anti-Caspase-3 (1:1000), anti-PARF (1:1000), anti-E-cadherin (1:500), anti-N-cadherin (1:500), anti-Vimentin (1:1000), anti-Snail (1:1000), anti-Lamin B (1:1000) (all from Cell Signalling Technologies, Danvers, MA, USA), anti-EIF4E-BP1 (1:1000), anti-phospho-EIF4E-BP1 (1:1000), anti-Wnt5β (1:1000), and anti-Twist (1:1000) (all from Abcam, Cambridge, MA) antibodies overnight at 4℃. Anti-β-actin antibody (1:5000; Sigma-Aldrich) was used as an internal control. Membranes were washed with TBST and incubated with horseradish peroxidase-conjugated secondary antibodies (Jackson Immunoresearch, West Grove, PA, USA) for 1 h at room temperature. After washing again with TBST, signal was detected using an enhanced chemiluminescence kit (Thermo Scientific, Rockford, IL, USA) and intensity was quantified using ImageJ software.

### Matrigel Invasion and wound healing assays

Matrigel invasion assay was performed using BD Biocoat Matrigel Invasion Chamber (pore size, 8 µm; 24 well; BD Biosciences, Bedford, MA, USA) according to the manufacturer's instructions. Briefly, overexpression cells, shEIF4E-BP1 or si*SRA* transfected cells, and siNC-transfected cells (5×10^4^ cells/mL) were plated into the upper chamber in serum-free medium while complete medium was added to the bottom chamber. After 48 h of incubation, cells that had invaded through the membrane were stained using a Differential Quik Stain kit (Diff Quik, Sysmex, Kobe, Japan). The assay was performed in triplicate.

Cell migration was assessed using monolayer wound healing assay. Briefly, cells were seeded into 6-well culture plates with serum-containing medium and allowed to grow to 90% confluency. The serum-containing medium was then removed, after which cells were serum starved for 24 h. When cell confluence reached nearly 100%, an artificial homogenous wound was created by scratching the monolayer with serum-free medium. Images of cells migrating into the wound were captured at 0, 24, and 48 h using a microscope. Each experiment was repeated three times.

### Cell viability assay

An equal number of cells (1×10^4^) transfected with siRNA, knocked down for EIF4E-BP1, and over-expressed with *SRA* were seeded into 96-well plates and incubated for 0 h, 24 h, 48 h, 72 h, and 96 h. The number of viable cells was determined using a Cell Counting Kit (CCK-8; Dojindo, Kumamoto, Japan). CCK-8 reagents were added to cultures and incubated for 4 h. The absorbance of each well was measured at wavelength of 450 nm with a micro-ELISA reader (Molecular Devices; Sunnyvale, CA, USA).

### Colony formation assay

ECC-1 and Ishikawa cells were seeded (50000 cells/well) in 6 well plates and incubated at 37℃ overnight. The following day, cells were incubated with siRNA, shRNA, or over-expression *SRA*. The culture medium was changed every week for another 2 weeks. Then, cells were washed twice with PBS, fixed with cold methanol for 30 min at 4℃ and stained with crystal violet dye (0.1% w/v) at room temperature for 1h. The plates were washed with water, dried and scanned.

### Flow cytometric analysis of apoptosis

For cell death analysis, after exponentially growing ECC-1 and Ishikawa cells were transfected with indicated plasmids for 48h, they were quantified by flow cytometry. Experiments were conducted using Annexin V-FITC Apoptosis Detection Kit (BD Pharmingen, San Diego, CA, USA) according to the manufacturer's instruction. Apoptosis was then analyzed by flow cytometry. Cells were sorted using a FACS LSR II (BD Biosciences) and analyzed with BD FACSDiva software version 6.2. Apoptotic cells were calculated after FACS analysis. The analysis was performed in triplicate.

### Immunofluorescence

ECC-1 cells were seeded onto glass coverslips in 24-well plates. The next day, cells were cultured in serum-free medium for 24 h and transfected with si*SRA* or *SRA* for overexpression for 48 h. Cells were fixed with cold methanol for 15 min and blocked with 5% normal rabbit serum and 0.4% Triton X-100 in PBS for 1 h. These cells were then incubated with primary antibodies of β-catenin (Cell Signaling, 1:500) at 4ºC overnight. Cells were then mounted using Vectashield hardset mounting medium with DAPI (Vector Laboratories, Burlingame, CA, USA). Photomicrographs were captured using a Confocal microscope (LMS700).

### Xenograft in mice

BALB/c nude mice (n = 10, 4-5 weeks of age, Orient Bio, Seongnam, Korea) were kept in aseptic conditions with constant temperature and humidity (Yonsei Medical University protocol). Each mouse received a subcutaneous injection of a 100 µL suspension of ECC-1 cells into the dorsal scapula area. Calipers were used to measure tumor size one time per 5 days. Tumor volume was calculated using a simplified equation to estimate a rotational ellipsoid (length × width^2^ × 0.5). Each tumor was harvested at 65 days post treatment.

### Magnetic resonance (MR) imaging and Micro PET imaging in mice

A Bruker Biospec 94/24 USR (9.4T) small animal scanner (35-mm diameter birdcage coil, Bruker BioSpin MRI, Ettlingen, Germany) was used to obtain MR images. T_2_-weighted images were obtained using rapid acquisition setting. They were acquired at the beginning inside the magnet bore. A 1.5% isoflurane and O_2_/N_2_O (1:1) mixture at flow rate of 0.7 L/min was used for anesthesia during MR experiment.

[^18^F]-fluorodeoxy-glucose (FDG) image was acquired as a reference to evaluate the agent as a diagnostic and therapy follow-up tracer. The same mouse was then injected with a ^124^I-la-beled derivative of pyropheophorbide-a, an imaging and photodynamic therapy bifunctional agent. Because of the long half-life of ^124^I (4.2 days), a longitudinal study (multiple scans over time) was possible with the same mouse and the same agent. Tumor uptake relative to the rest of the body increased over time, indicating that the agent had promising potential as both a therapeutic and a tumor-monitoring agent. After incubation with FDG, endometrial tumors were imaged using a microPET scanner (Inveon™ Dedicated PET, Siemens). PET image analyses were then performed using a standard software (Inveon™ Acquistion workplace).

### Hematoxylin and eosin (H&E) staining

Mouse were sacrificed. Tumor tissues were collected, fixed in 4% paraformaldehyde for 24 h, washed in PBS, and then embedded in paraffin. Two-micrometer sections were stained with hematoxylin and eosin following standard procedures.

### Statistical analysis

Results are expressed as mean ± standard deviation (SD) or mean ± standard error of mean (SEM). All statistical analyses were performed using SPSS standard version 20.0 (IBM, Chicago, IL, USA). Pearson's χ2 test, Student's *t*-test, and Fisher's exact test were used to evaluate associations of *SRA* with clinicopathological characteristics. The Kaplan-Meier method was used to analyze overall survival time. Log-rank test was used to estimate differences between groups. Stepwise Cox regression model was used for multivariate survival analysis of parameters that were significant in univariate analysis. All tests were two-sided and a *P* value < 0.05 was considered to indicate statistically significant result.

### Ethics approval and consent to participate

This study was approved by the Ethics Committee of Yonsei Severance Hospital, and informed consent was obtained from all patients. Samples were collected from 146 endometrial cancer patients who underwent surgery and a control group of 57 patients with benign gynecologic disease between Sep 2012 and Dec 2014.

## Results

### Expression of *SRA* is Upregulated in EC Tissues

**Figure 1:**
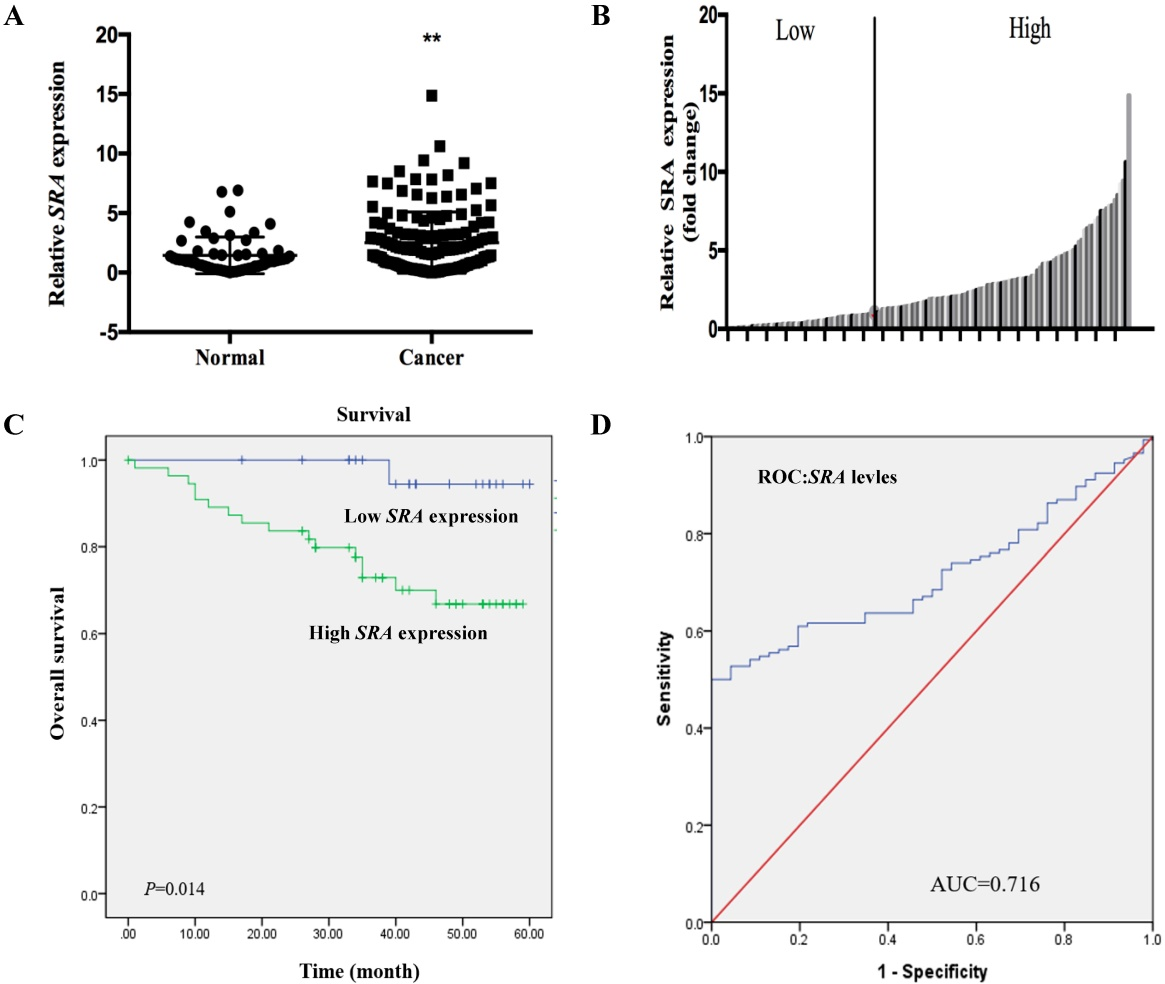
***SRA* expression in human endometrial cancer patient tissues.** (A) Relative expression of *SRA* was significantly higher in endometrial cancer (EC) patient tissues (n=146) than that in noncancerous patient tissues (n=57). *SRA* expression was determined using quantitative real time polymerase chain reaction with β-actin as an internal control. (B) *SRA* expression was classified into two groups according to the expression level of EC tissues. (C) Relative *SRA* expression and its clinical significance based on Kaplan-Meier overall survival curves of patients with endometrial cancer and different levels of *SRA*. (D) Receiver operating characteristic (ROC) curve for prognosis prediction of patients using *SRA* level. The area under curve (AUC) is shown in plots. Data are expressed as mean ± standard deviation. *p<0.05 vs. non-tumor control.

Expression levels of *SRA* in 146 EC patient tissues and 57 corresponding normal endometrial tissues were determined by real time-PCR and normalized to β-actin. *SRA* expression in EC tissue was more than 2.9-fold higher than that in non-cancerous tissue (*p* < 0.05) (Figure 1A). Clinicopathologic factors and patient survival were compared between high (n = 92) and low *SRA* expression groups (n = 54) (Figure 1B). Clinicopathological data such as age, stage, histologic grade, BMI, tumor size, menopause, and lymph node metastasis were compared between high and low *SRA* expression groups (Table 1). High grade histology, tumor size, and lymph node metastasis were more frequently found in the high *SRA* expression group (*p* < 0.05). With regard to overall survival, patients with high *SRA* expression had significantly poorer prognosis than those with low *SRA* expression (Figure 1C). Furthermore, receiver operating characteristic curve analysis showed that *SRA* level was useful for predicting survival of EC patients (area under the curve: 0.716; 95% confidence interval [CI]: 0.645 to 0.786) (Figure 1D). Univariate analysis of overall survival revealed that the relative level of *SRA* expression, age, histologic grade, tumor size, menopause, and lymph node metastasis were prognostic indicators (Table 2). Variables with a value of *p* < 0.05 were selected for multivariate analysis. Multivariate analysis showed that *SRA* expression, age, and grade were independent prognostic indicators for overall survival in patients with EC (Table 2).

### *SRA* knockdown decreases proliferation, migration, and invasion of EC cells

**Figure 2:**
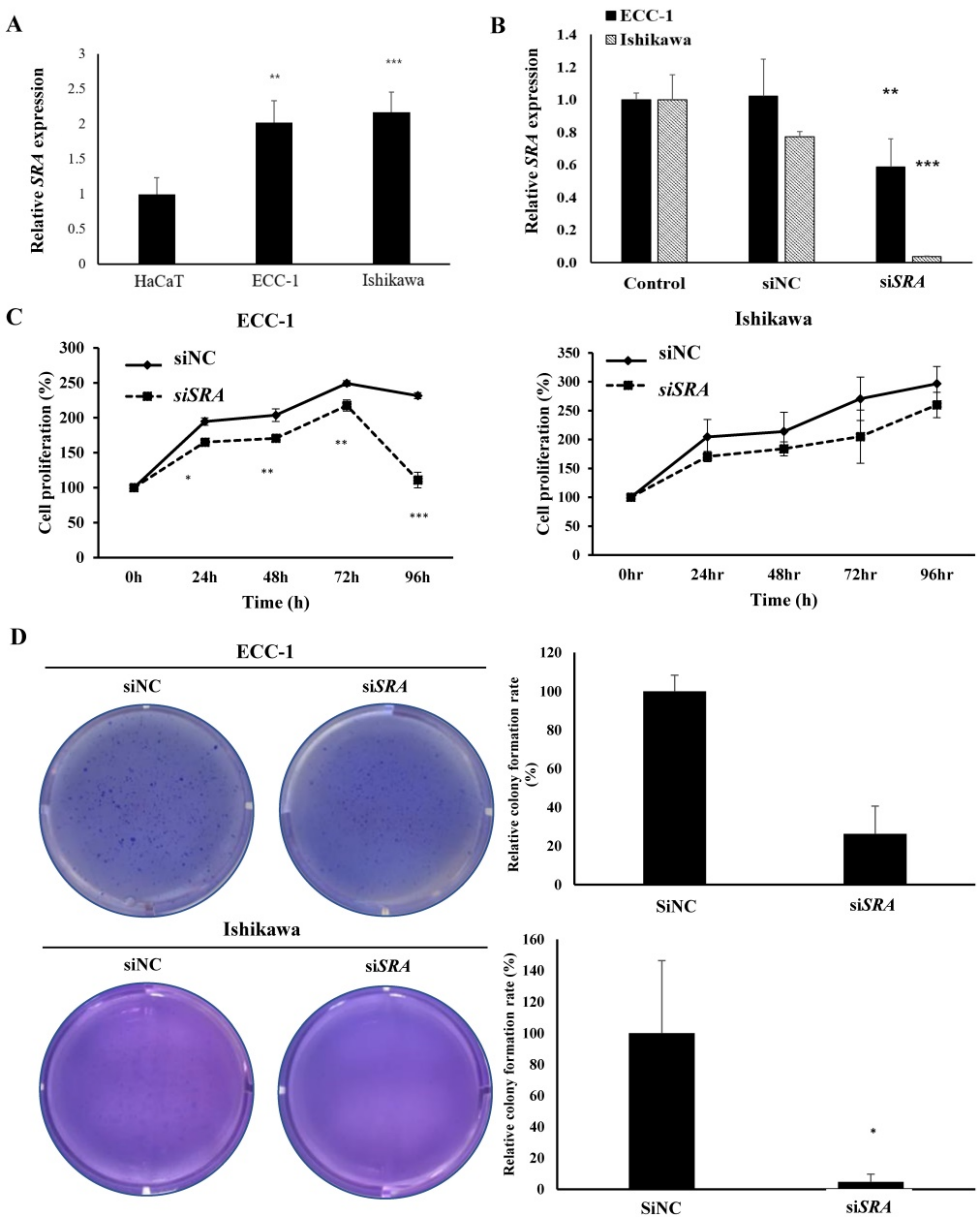

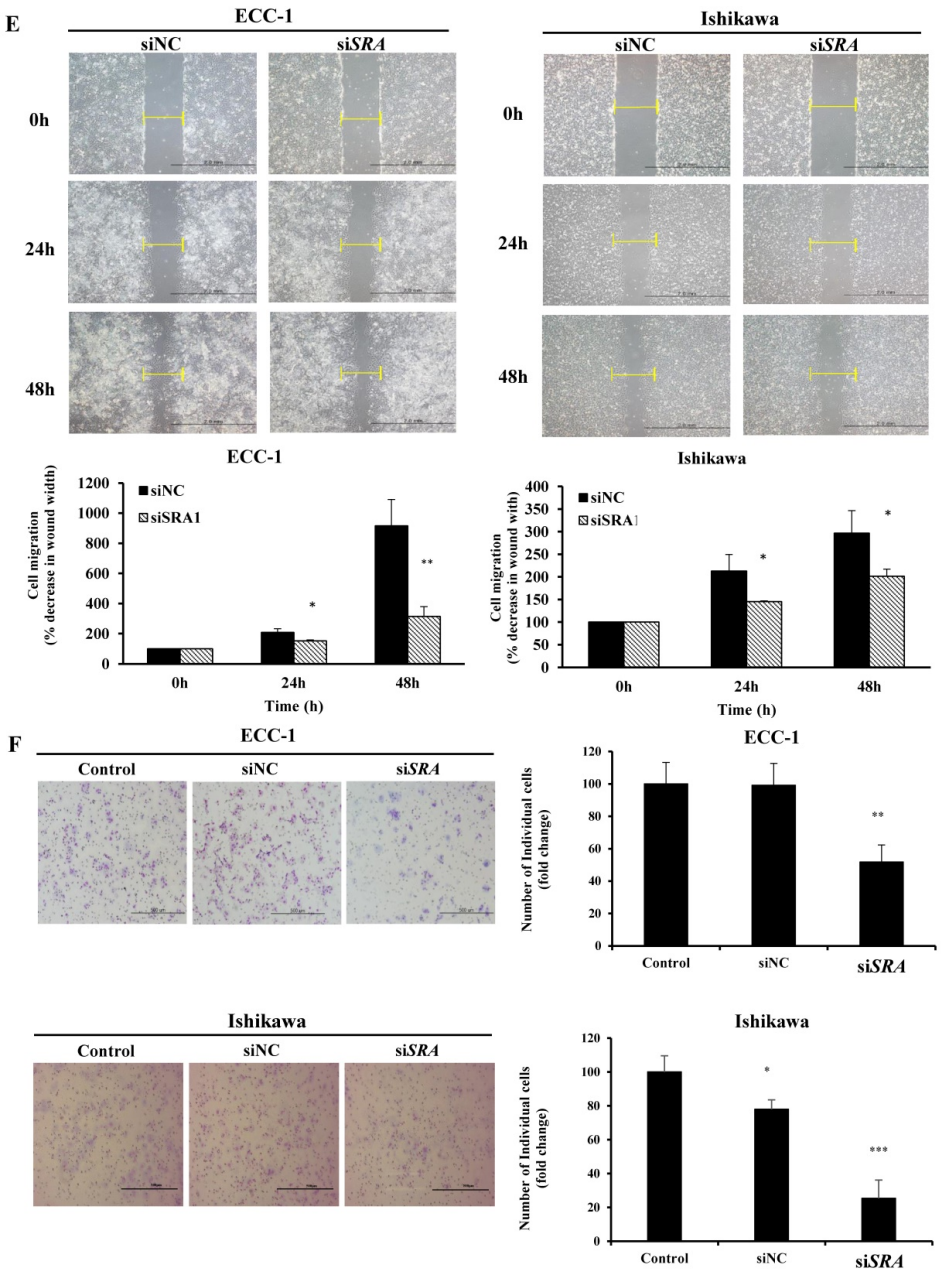
**Knockdown of *SRA* inhibits endometrial cancer cell proliferation, migration, and invasion.** (A) Expression of *SRA* in human keratinocyte (HaCaT) and human endometrial cancer cell lines determined by quantitative real time polymerase chain reaction (qRT-PCR). (B) Knockdown efficiency was determined by qRT-PCR analysis in ECC-1. Cells were transfected with *SRA* siRNA (si*SRA*) or negative control siRNA (siNC). (C) Knockdown of *SRA* decreases cell proliferation in ECC-1 and Ishikawa cells. The proliferation of endometrial cancer cells transfected with si*SRA* and negative control siRNA (siNC) was determined using Cell Counting Kit-8 assays (siRNA: 10µM). (D) Knockdown of *SRA* inhibited colony formation in ECC-1 and Ishikawa cells. (E) Wound healing assay was used to determine migration in *SRA*-specific siRNA (si*SRA*)-transfected ECC-1 and Ishikawa cells (×200). (F) Matrigel invasion assay was used to determine invasion after 48 h in si*SRA* transfected ECC-1 and Ishikawa cells. Bars indicate mean ± standard deviation of three independent experiments performed in triplicate. *p < 0.05, **p < 0.01, and ***p < 0.001 vs. siNC.

To investigate the role of *SRA* in EC cell lines, ECC-1 cells and Ishikawa cells were examined for *SRA* expression. Results showed that ECC-1 cells and Ishikawa cells expressed higher levels of *SRA* than control cells (HaCaT) (Figure 2A). Knockdown efficiency of *SRA* specific siRNAs (si*SRA*) was analyzed by qRT-PCR. Results revealed that si*SRA* had higher silencing efficiency compared to control (siNC) (Figure 2B). The proliferation of si*SRA*-transfected EC cells and siNC was measured by CCK-8 assay. At 96 h post-transfection, knockdown of *SRA* inhibited cell proliferation by 55% and 10% in ECC-1 and Ishikawa cells, respectively, relative to control (siNC) (Figure 2C). In addition, knockdown of *SRA* inhibited colony formation in ECC-1 and Ishikawa cells (Figure 2D). Effects of *SRA* on invasion and migration of EC cells were assessed by wound healing assays and Matrigel invasion, respectively. Wound healing assays showed larger width of wound in si*SRA*-transfected ECC-1 and Ishikawa cells than that in siNC-transfected cells, demonstrating decreased migration of EC cells via down-regulation of *SRA* (Figure 2E). According to Matrigel invasion assay, knockdown of *SRA* significantly reduced the number of invasive cells by more than 53% in ECC-1 cells (Figure 2F). We repeated with another siRNA to a different region of the transcript to minimize the possibility of off-target effects. The most efficient si*SRA*1 was selected and used for the experiment (Supplementary Figure 1).

### Overexpression of *SRA* promotes proliferation, migration, and invasion of EC cells

**Figure 3:**
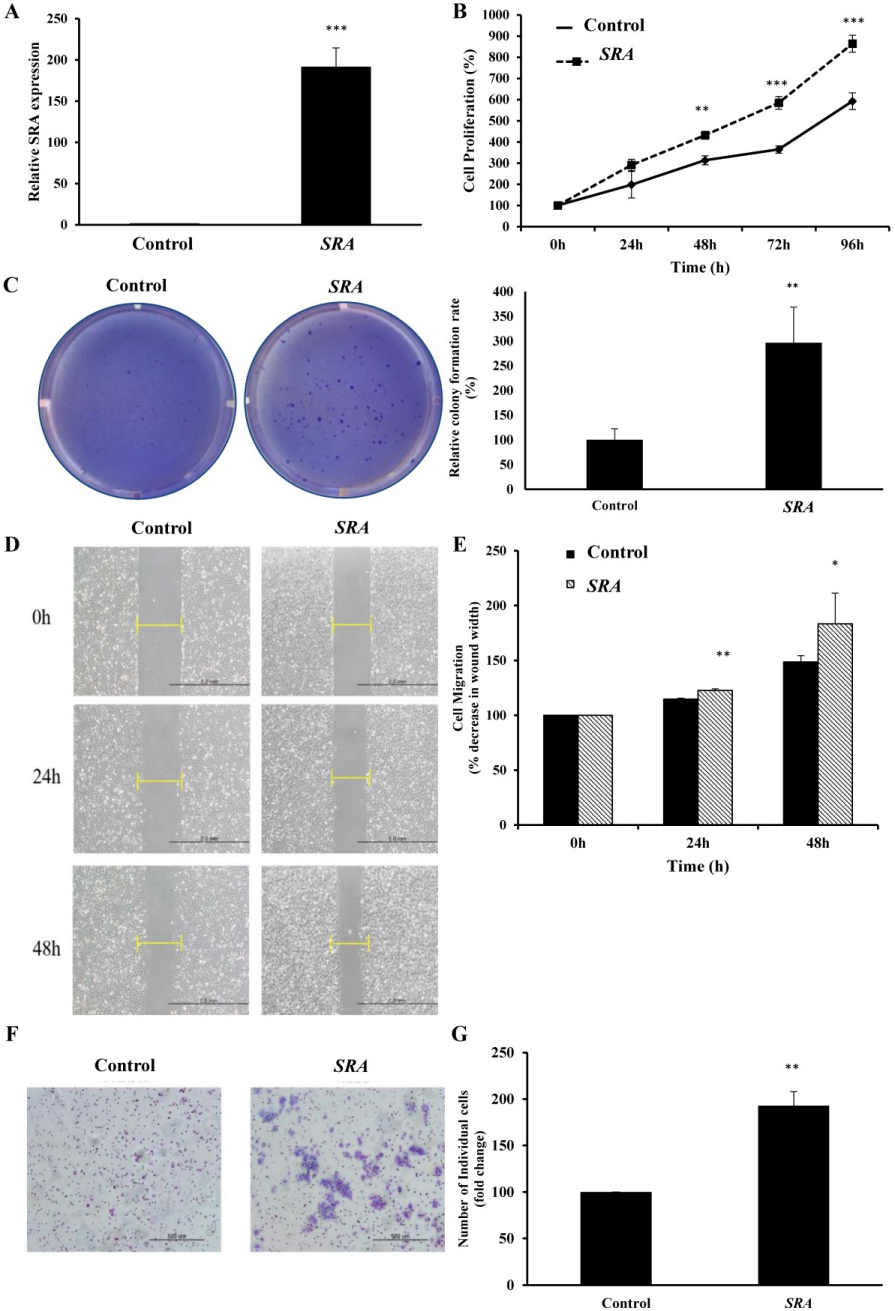
** Overexpression of *SRA* inhibits endometrial cancer cell proliferation, migration, and invasion.** (A) Overexpression of *SRA* in ECC-1 cells was analyzed using qRT-PCR. (B) Cell proliferation was analyzed using Cell Counting Kit-8 assays. (C) Overexpression of *SRA* promoted cell colony formation in ECC-1 cells. (D) Wound healing assay observed under the optical microscope was used to determine migration in *SRA*-overexpressing ECC-1 cells (×200). Cells after 24 and 48 h of incubation were analyzed and determined using ECC-1 cells as control. (E) Wound healing assay results showing percentage of each cell line. (F) Cell invasion observed under an optical microscope. Matrigel invasion assays were used to determine invasion after 48 h of incubation of *SRA*-overexpressing ECC-1 cells. (G) Matrigel invasion assay results showing percentage of each cell line. Bars indicate mean ± standard deviation of three independent experiments performed in triplicate. *p < 0.05, **p < 0.01, and ***p < 0.001 vs. ECC-1, Vector cells.

Lentiviral-mediated overexpression of *SRA* was performed to determine the functional role of this lncRNA in ECC-1 cells. qRT-PCR analysis showed that *SRA* was successfully overexpressed in ECC-1 cells compared to that in control cells (*p* < 0.001) (Figure 3A). We next examined the impact of *SRA* overexpression on cell proliferation. Results of CCK-8 assays showed that overexpression *SRA* in ECC-1 cells increased cell proliferation (Figure 3B), suggesting that *SRA* was involved in the proliferation of EC cells. Thereafter, Colony formation assays confirmed that *SRA* overexpression promoted cell colony formation (Figure 3C). Effects of *SRA* on invasive and migratory behaviors of cells were assessed by wound healing assays and Matrigel invasion, respectively. Overexpression of *SRA* resulted in increased migration of ECC-1 cells relative to empty vector-expressing controls (Figure 3D). There was a significant difference between scratch width percentages of each cell line at 24 and 48 h after scratching. Empty vector and *SRA* overexpression groups of ECC-1 cells were significantly different in scratch width (Figure 3E). Furthermore, *SRA* overexpression in ECC-1 cells significantly increased invasion relative to that in empty vector-expressing cells. Relative percentages of invaded cells of ECC-1 at 48h after incubation in empty vector and *SRA* overexpression groups were significantly different (Figure 3F). Taken together, these results indicate that overexpression of *SRA* can promote invasion and migration of ECC-1 cells *in vitro*.

### Effect of lncRNA *SRA* on Wnt/ β-catenin signaling pathway

**Figure 4:**
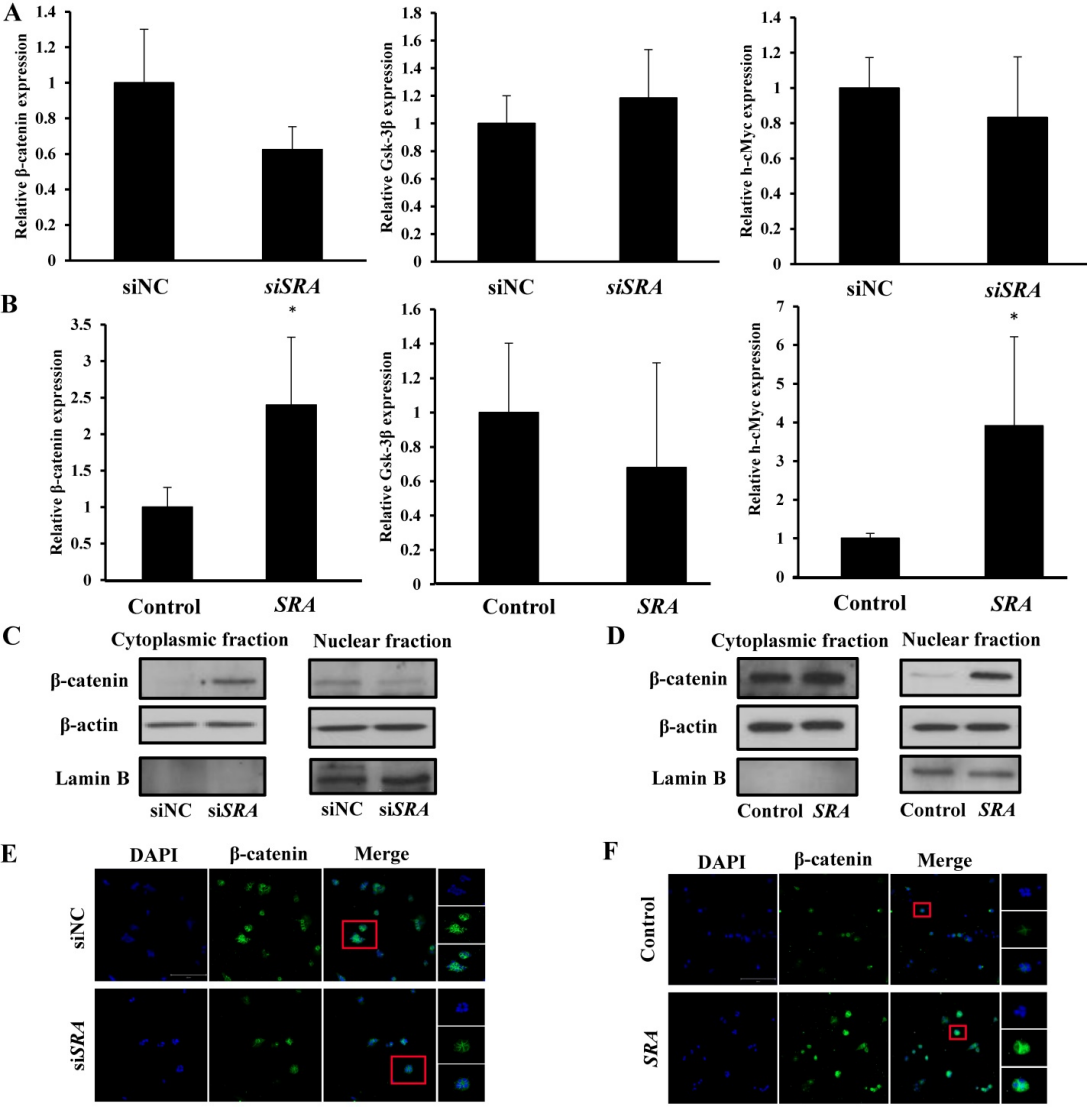
** Wnt/β-catenin pathway mediates oncogenic activity of *SRA* in ECC-1 cells.** (A) Expression levels of β-catenin, Gsk-3β, and h-cMyc mRNAs were measured in ECC-1 cells transfected with si*SRA* or siNC. (B) Expression levels of β-catenin, Gsk-3β, and h-cMyc mRNAs were measured in *SRA* over-expressing ECC-1 cells. Bars indicate mean ± standard deviation of three independent experiments performed in triplicate. *p < 0.05 vs. siNC. (C) Cytoplasmic and nuclear β-catenin levels in *SRA* knockdown ECC-1 cells were measured. (D) Cytoplasmic and nuclear β-catenin levels in *SRA* over-expressing ECC-1 cells were measured. (E, F) Subcellular β-catenin localization in indicated cells was assessed by immunofluorescence staining. Immunofluorescence staining of β-catenin (green) showed nuclear and cytoplasmic localization in cells transfected with siNC, si*SRA*, empty vector (control), or *SRA* over-expression. Nuclei were counterstained with DAPI (blue). Total magnification was 200×. Images were zoomed in 500%.

Previous studies have suggested that *SRA* is involved in cell proliferation, invasion, migration, and metastasis. 24 However, the mechanism through which that *SRA* plays a role in EC has not been elucidated yet. The Wnt signaling pathway regulates various developmental processes such as cell migration, attachment, proliferation, and cell death. In the present study, we tried to confirm the functional relationship between Wnts and EC. We evaluated expression levels of β-catenin, Gsk-3β, and h-cMyc. Results showed that β-catenin and h-cMyc expression levels were decreased whereas Gsk-3 β levels were increased in the group transfected by si*SRA* compared to those in the group transfected by siNC (Figure 4A). Moreover, β-catenin and h-cMyc expression levels were increased whereas Gsk-3 β levels were decreased by overexpression of *SRA* compared with those in the control transfected with an empty vector) (Figure 4B). We also evaluated protein levels of β-catenin. Results showed that its expression was markedly decreased in the group transfected with si*SRA* (compared to that in the group transfected with siNC) but increased in the group overexpressed with *SRA* (compared to that in the group transfected with an empty vector) (Figure 4C, 4D). Next, we used cell fractionation method to examine β-catenin distribution in the nucleus and cytoplasm. We found that cytoplasmic β-catenin was substantially increased in *SRA*-silenced ECC-1 cells compared to that in control cells transfected with siNC. On the contrary, the reserve result was observed for nuclear β-catenin (Figure 4E). In addition, we also found that cytoplasmic β-catenin was substantially decreased in *SRA* over-expressed ECC-1 cells compared to that in control cells transfected with an empty vector. The reverse result was observed for nuclear β-catenin (Figure 4F). Taken together, these data suggest that gene silencing of *SRA* can inactivate the Wnt/ β-catenin signaling pathway.

### *SRA* interacts with EIF4EBP1

**Figure 5:**
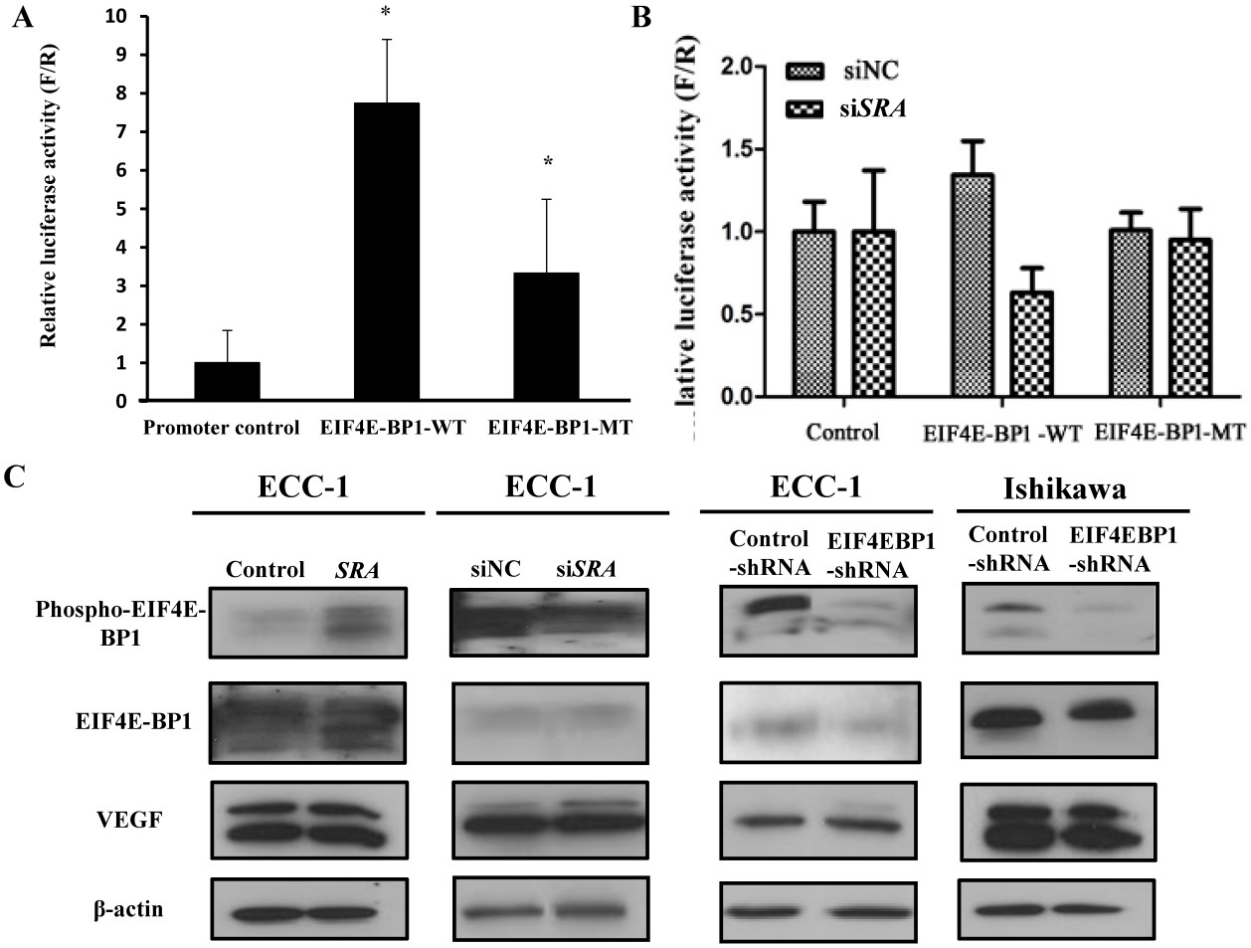
** Luciferase assay of EIF4E-BP1 promoter activity in ECC-1 cells.** (A) The negative pGLO.4 insert indicates an empty sequence. Data are presented after normalizing transfection efficiency using Renilla luciferase reporter gene. (B) ECC-1 cells were co-transfected with indicated luciferase reporter plasmids, siNC, or si*SRA*-EIF4E-BP1. Luciferase activity was analyzed 24 h later. Bars indicate mean ± standard deviation of three independent experiments performed in triplicate. *p < 0.05 vs. promoter control. (C) p-EIF4E-BP1, EIF4E-BP1, and VEGF expression was analyzed by western blotting.

To validate the interaction between *SRA* and EIF4E-BP1, luciferase assays were carried out. We constructed luciferase reporter plasmids for EIF4E-BP1 containing predicted wild-type and mutant-binding sites for EIF4E-BP1 (Figure 5A). We found that downregulation of *SRA* reduced the luciferase activity of the wild-type plasmid but not that of the mutant plasmid. Co-transfection of the plasmid containing the wild-type UTR plus an EIF4E-BP1, but not a negative control, resulted in a significant increase in relative luciferase activity (Figure 5B). In contrast, co-transfection of the plasmid with the mutated UTR plus either mimic completely transfected si*SRA* repression, demonstrating the specificity of these si*SRA* for EIF4E-BP1 suppression. In addition, p-EIF4E-BP1, EIF4E-BP1, and VEGF expression levels were analyzed by western blotting. In ECC-1 cells, *SRA* knockdown and *SRA* overexpression regulated EIF4E-BP1 expression typical of tumor progression. The same results were obtained for cells transfected with EIF4E-BP1 (Figure 5C). Expression levels of phospho-EIF4E-BP1 and EIF4E-BP1 were increased in *SRA*-overexpressed cells but decreased in *SRA* knocked-down cells. Expression levels of phosphor-EIF4E-BP1 and EIF4E-BP1 were also decreased in cells transfected with shEIF4E-BP1. There was no significant difference in the expression of VEGF.

### Knockdown of EIF4E-BP1 suppresses proliferation, migration, invasion, and apoptosis of EC cells

**Figure 6:**
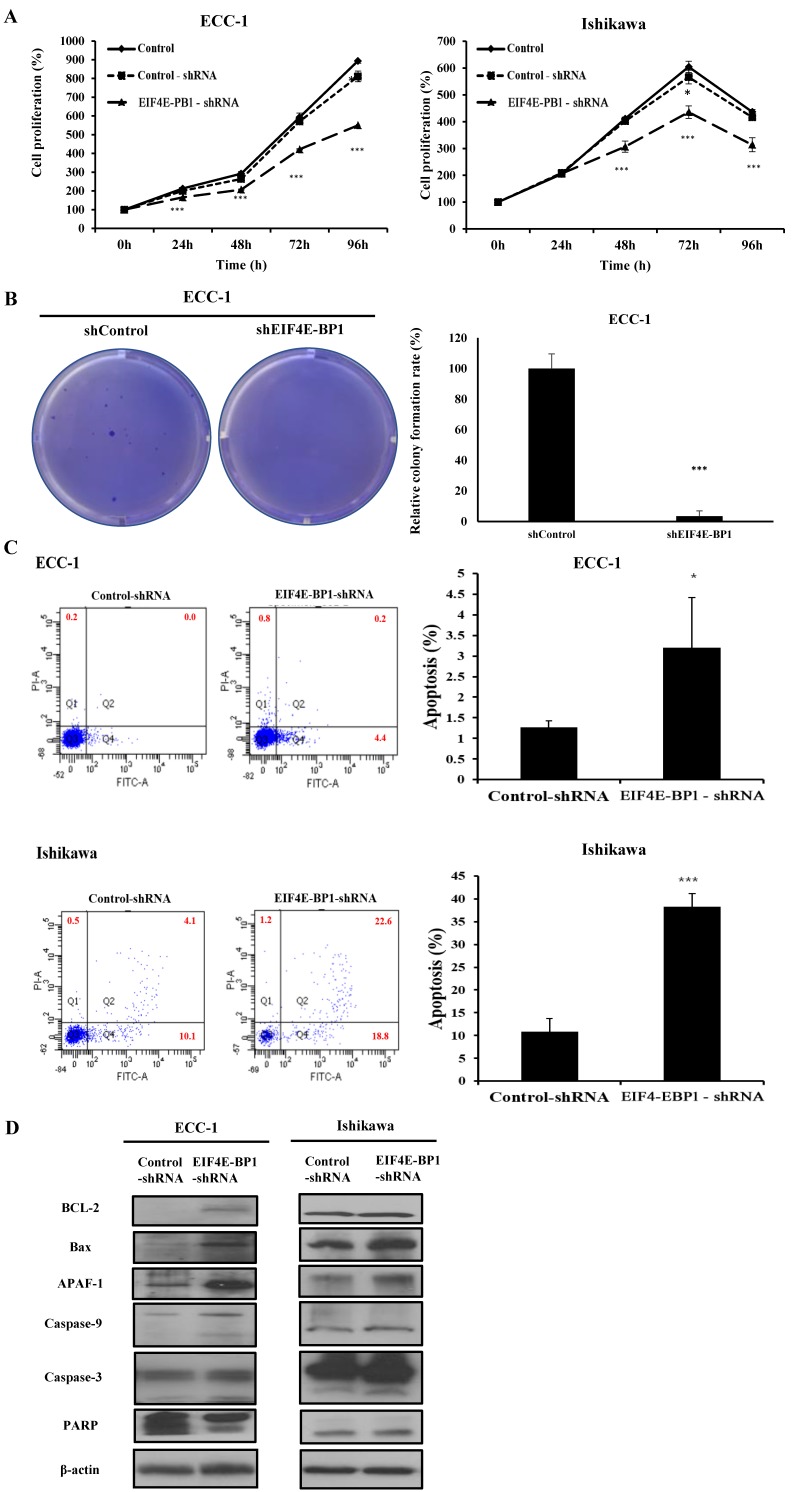

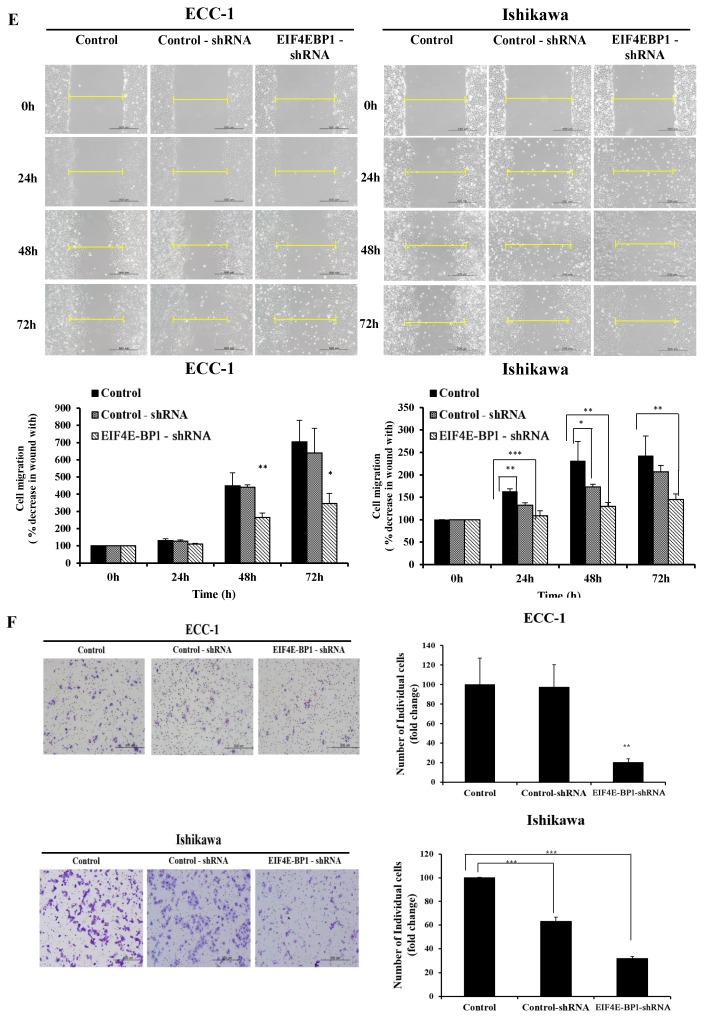
** Knockdown of EIF4E-BP1 decreased the proliferation and apoptosis of endometrial cancer cells.** (A) Viability of ECC-1 and Ishikawa cells transfected with NC-shRNA or EIF4E-BP1 based on CCK-8 assay. (B) Down-regulation of EIF4E-PB1 inhibited cell colony formation in ECC-1 cells. (C) Apoptosis of ECC-1 and Ishikawa cells was detected by flow cytometry. (D) Western blot was used to assay protein levels of BCL-2, Bax, APAF-1, cleaved Caspase 3/9, and PARP in endometrial cancer cells transfected with NC-shRNA or EIF4E-BP1-shRNA. (E) Wound healing assay for the migration of ECC-1 and Ishikawa cells transfected with NC-shRNA or EIF4E-BP1-shRNA. (F) Matrigel invasion assay for the invasion of ECC-1 and Ishikawa cells. Bars indicate mean ± standard deviation of three independent experiments performed in triplicate. *p < 0.05, **p < 0.01, and ***p < 0.001 vs. control or NC-shRNA.

To further determine whether EIF4E-BP1 could regulate *SRA* expression, the expression of EIF4E-BP1 was knocked down in ECC-1 and Ishikawa cells. We repeated with another siRNA to a different region of the transcript to minimize the possibility of off-target effects. (Supplementary Figure 2). After confirming the effects of siEIF4E-BP1, the EIF4E-BP1-shRNA cell line was used in the experiment. Knockdown of EIF4E-BP1 was performed by transfecting with EIF4E-BP1-shRNA. Viability, migration, invasion, and apoptosis of EC cells were then determined using CCK-8, wound healing assays, Matrigel invasion, and flow cytometry, respectively. We found that downregulation of EIF4E-BP1 notably inhibited cell viability (Figure 6A). In addition, downregulation of EIF4E-BP1 significantly inhibited colony formation in ECC-1 cells (Figure 6B). We further investigated the potential effect of EIF4E-BP1 on EC cell apoptosis. Results of flow cytometry analysis determined by Annexin V-FITC/PI double staining revealed that knockdown of EIF4EPB1 significantly induced apoptosis of ECC-1 and Ishikawa cells compared with the control (transfection with NC-shRNA) (Figure 6C). Besides, downregulation of EIF4E-BP1 notably elevated levels of BCL-2, Bax, APAF-1, cleaved Caspase 3/9, and PARP in EC cells (Figure 6D). We further investigated whether EIF4E-BP1 could affect the migration and invasion of EC cells. Wound healing assay showed that the migration ability was significantly decreased in the EIF4E-BP1-shRNA group compared to that in the NC-shRNA group both in ECC-1 and Ishikawa cells (Figure 6E). The result of Matrigel invasion assay revealed that knockdown of EIF4E-BP1 remarkably reduced the number of invasive cells (Figure 6F).

### Overexpression of *SRA* in ECC-1 cells increases xenograft tumor growth *in vivo*

**Figure 7:**
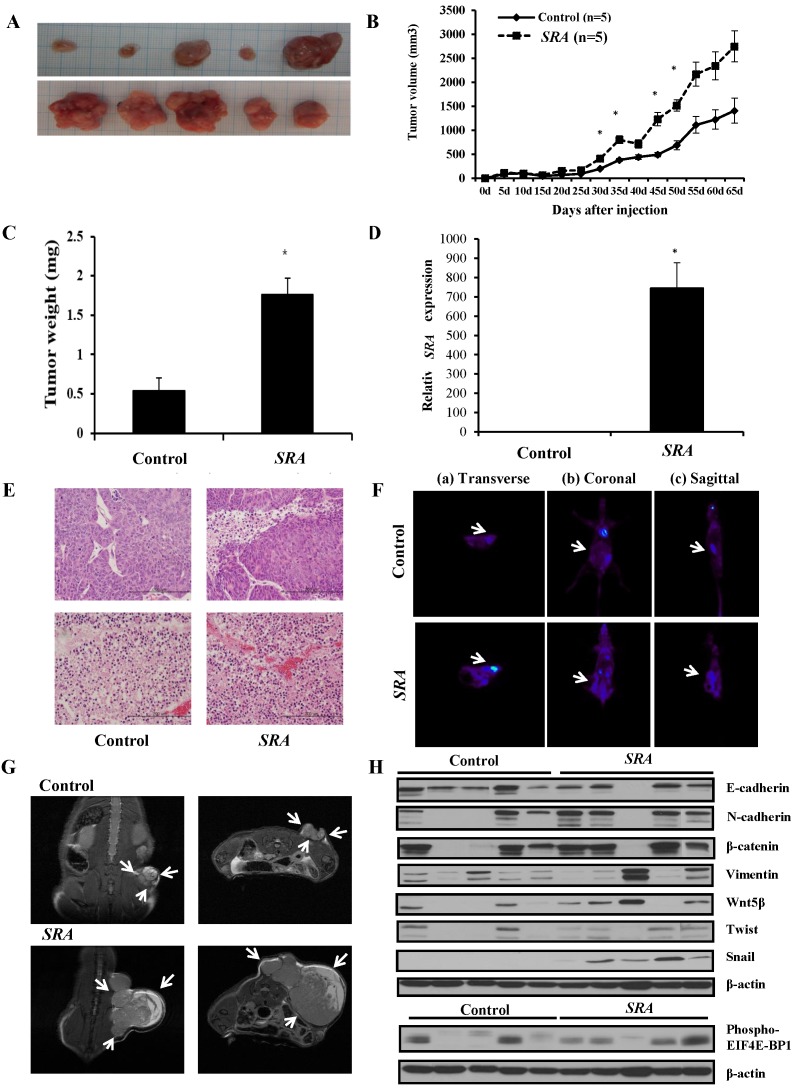
** Effect of *SRA* on tumor growth *in vivo*.** (A) ECC-1 cells (5 × 10^6^) stably expressing *SRA* were inoculated into nude mice and the effect of *SRA* on endometrial cancer growth was examined after 65 days (n=5). Photograph of tumors are presented. (B) Tumor volume was calculated every 5 days. Data are presented as mean ± SD (n=5). * *p*< 0.05 vs. control. (C) Tumor weight. Data are presented as mean ± SD. * *p*< 0.05 vs. control. (D) qRT-PCR analysis of *SRA* expression in tissues of resected tumors. Bars indicate mean ± standard deviation of three independent experiments performed in triplicate. **p < 0.01 vs. Control. (E) Haematoxylin and eosin (H&E) staining at 65 days after injection. (F) Micro PET image with transverse (a), coronal (b), and sagittal (c) plane slices of mice showing FDG uptake in the affected right carotid artery (arrows). (G) MRI imaging. (H) *SRA* overexpression promotes EMT, Wnt/β-catenin and mTOR-related expression in xenograft. E-cadherin, N-cadherin, β-catenin, Vimentin, Wnt-5β, Twist, Snail, and 4EBP1 expression levels were analyzed by western blotting.

*In vitro* studies showed that overexpression of *SRA* promoted EC cells proliferation, invasion, and migration. To further determine whether *SRA* expression could enhance the tumorigenicity of EC ECC-1 cells *in vivo*, we inoculated ECC-1 cells as xenografts into nude mice (Figure 7A). Tumor volume and weight were measured. Mean tumor volumes and weights (2752.214 ± 326.1 mm^3^ and 1,760 ± 20 mg, respectively) at day 65 in mice receiving *SRA*-overexpressing ECC-1 cells were significantly larger than those (1409.166 ± 260.1 mm^3^ and 542 ± 16 mg, respectively) in mice receiving empty vector-expressing cells (*p* < 0.05) (Figure 7B). *SRA* expression in tumor tissue was significantly greater in *SRA*-overexpressing cells compared to that in control cells (Figure 7C, 7D). Tumor weight was correlated with tumor volume as determined by microscale (*p* < 0.05). Histological examination revealed that more cells with large nucleoli and irregular nuclear membranes were present in *SRA*-overexpressing xenografts than those in control xenografts (Figure 7E). We further evaluated tumor size and activity using magnetic resonance imaging (MRI) and positron emission tomograph (PET) (Figure 7F, 7G). Tumor growth was strongly induced by *SRA* overexpression. Tumor size and fluorodeoxyglucose (FDG) accumulation were significantly larger and greater, respectively, in mice inoculated with *SRA*-overexpressing cells. These findings suggest that *SRA* could promote tumor growth *in vivo*, further supporting our hypothesis that *SRA* is involved in the pathogenesis of malignant transformation of EC cells. Next, we determined expression levels of Wnt/ β-catenin related proteins in xenografts derived from *SRA*-overexpressing ECC-1 cells. E-cadherin, N-cadherin, β-catenin, Vimentin, Wnt-5β, Twist, Snail, and EIF4E-BP1 protein levels were greater in *SRA*-overexpressing tumor than those in control tumors (Figure 7H).

## Discussion

Recently, lncRNA has become the focus of intensive research because it plays an important role in malignant processes including tumor formation, drug resistance, and metastasis. 25, 26 LncRNA exhibits tissue-specific expression patterns. It has been functionally characterized. Biosynthesis of theses RNAs is important for a variety of physiological processes. Abnormal expression of lncRNA may affect cancer development and progression. 17 However, the molecular mechanism of lncRNA associated with tumor progression and metastasis has not been fully understood yet.

Previous studies have examined molecular function and clinical significance of SRA expression in cervical cancer tissues and cell lines. Transitional effects of SRA appear to be mediated, at least in part, by regulation of genes involved in cell migration, invasion and EMT. 15, 24 However, little is known about the biological function of SRA in EC. Also, since molecular mechanisms of lncRNA associated with tumor progression and metastasis are not fully understood yet, questions have been raised as to whether *SRA* promotes metastasis of EC by modulating gene expression encoding proteins involved in metastasis. 27 A deeper understanding of the molecular mechanism underlying the progression and metastasis of EC is essential for the development of more effective therapies and for the identification of new diagnostic markers for EC.

Most of previous studies were focused on *SRA* gene expression. In this study, the expression of *SRA* gene was increased in 63% of EC patients and correlated with FIGO stage and lymph node metastasis. High expression of the *SRA* gene was positively correlated with overall survival of EC. Furthermore, knockdown of *SRA* inhibited proliferation, invasion, and migration of EC cells. Conversely, overexpression of *SRA* increased proliferation, invasion, and migration of ECC-1 cells, suggesting that *SRA* could contribute to the invasiveness and mobility phenotype of EC cells. These observations are relevant to *SRA* because its upregulation is similarly associated with cancer cell growth and migration.

Next, we investigated the mechanism of *SRA* by *SRA* target prediction. One possible mechanism is through Wnt signaling. It is known that Wnt family regulates a wide range of cellular functions, including cell growth, proliferation, polarity, differentiation, and development. 28, 29 As an important transcription factor, β-catenin acts as a major effector of the canonical Wnt signaling cascade. 30 As a result, we found that *SRA* regulates β-catenin, Gsk-3β, and h-cMyc involved in the activation of the Wnt/ β-catenin signaling pathway. In addition, we hypothesized that the expression of *SRA* could regulate the Wnt signaling pathway. Cytoplasmic β-catenin is degraded by multiple proteolytic complexes when the Wnt signaling pathway is inactive. Conversely, β-catenin avoids degradation and accumulates in the cytoplasm. It eventually translocates to the nucleus during Wnt signaling pathway activity and displays transcriptional activity. 31 As expected, the amount of β-catenin was significantly decreased in the nucleus after *SRA* knockdown but increased in the nucleus after *SRA* overexpression. The Wnt/ β-catenin signaling pathway is known to regulate gene expression through cyclin D1 and c-Myc. 32 Eukaryotic translation initiation factor 4E-binding protein 1 (EIF4E-BP1) mediates a tight regulation of expression of many proteins including cyclin D1, survivin, c-Myc, and vascular endothelial growth factor (VEGF) that are critical to cell division, cell growth, and angiogenesis. 33

In case studies, EIF4E-BP1 alteration was strongly related to survival in ovarian cancer patients. 34 EIF4E-BP1 mediates phenotypic changes by selectively enhancing translation of restricted mRNA pools encoding proteins associated with malignant tumors. This enhanced EIF4E-BP1 can contribute to all aspects of malignant progression. 35 Post-transcriptional inhibition of EIF4E-BP1 mediated pathway in EC cells by *SRA* was demonstrated in this study. We found a direct interaction between *SRA* and EIF4E-BP1. Knockdown of *SRA* was associated with inhibition of luciferase activity under the control of EIF4E-BP1 3'-UTR. In addition, it has been demonstrated that *SRA* knockdown induces significant downregulation of EIF4E-BP1 mRNA and protein. Our results showed that EIF4E-BP1 was a direct target of SRA and that the reduction of SRA partially reduced EIF4E-BP1 expression as well as invasiveness and mobility. EIF4E-BP1 shRNA reduced cell proliferation and invasion in ECC-1 and Ishikawa cells. This indicates that this ability is EIF4E-BP1-denpendent.

In conclusion, our study emphasizes the clinical validity of *SRA* in predicting the prognosis of EC. It provides homeostasis and interactions in the EC through the Wnt/ β-catenin signal. Further experiments revealed that EIF4E-BP1 was a direct and functional target of *SRA* in EC cells. Thus, *SRA* can be a diagnostic marker and therapeutic target in the treatment of EC.

In summary, steroid receptor RNA activator (SRA) is a long non-coding RNA with an important function in tumor. It can activate human hormone receptors that are strongly associated with gynecologic cancers. In addition, *SRA* knockdown can inhibit the expression of EIF4E-BP1 that plays a critical role in the control of protein synthesis, cell growth, and cell survival, thus promoting tumorigenesis and epithelial-mesenchymal transition (EMT) that are important for cell motility and metastasis. Results of this study indicate that lncRNA *SRA* is an important feature directly related to disease progression, death, and recurrence as well as endometrial cancer tumor growth, metastasis, EMT, and multiple regulators. These findings indicate that *SRA* may represent a novel biomarker for predicting recurrence and prognosis. It can serve as a promising therapeutic target in endometrial cancer.

## Supplementary Material

Supplementary figures and tables.

## Figures and Tables

**Table 1:** Association between *SRA* expression and clinicopathologic factors in endometrial cancer (n=146).

		*SRA* expression		
	n (%)	Low	High	*P*-value^a^
Age (mean±SD)	146	52.49±7.41	51.99±9.93	0.749
Stage				0.001
Ι	101	47	54	
Ⅱ	13	1	12	
Ⅲ	19	3	17	
Ⅳ	13	3	10	
Grade				0.258
Ι	75	32	43	
Ⅱ	57	17	40	
Ⅲ	14	4	10	
BMI				0.185
<18.5	6	1	5	
18.5_25	74	33	41	
>25_30	55	16	39	
>30	11	3	8	
Tumor size				0.413
<2cm	60	26	34	
2-3.9cm	35	11	24	
4-5.9cm	15	6	9	
≥6cm	36	10	26	
Menopause				0.715
No	44	15	29	
Yes	102	38	64	
Lymphatic metastasis				0.025
No	129	51	78	
Yes	17	2	15	

^a^Chi-square test or Fisher's exact test were used to calculate P-values.

**Table 2:** Univariate and multivariate analysis of various factors for overall survival.

	Overall Survival			
	Univariate analysis		Multivariate analysis	
	HR (95% CI)	*P*	HR (95% CI)	*P*
*SRA* expression	11.114 (1.481-83.412)	0.003	10.218 (1.340-77.916)	0.025
Age, years (continuous)	1.102 (1.051-1.156)	0.0001	1.107 (1.042-1.175)	0.001
FIGO stage	2.171 (1.499-3.143)	0.000004	1.507 (0.948-2.395)	0.083
Grade	3.066 (1.611-5.834)	0.0003	2.480 (1.137-5.409)	0.022
Lymph node metastasis	3.009 (1.081-8.375)	0.027	0.937 (0.251-3.494)	0.922
BMI	0.806 (0.404-1.604)	0.538		
Menopause	3.494 (0.807-15.138)	0.074		
tumor size	1.521 (1.058-2.186)	0.018	0.955 (0.628-1.452)	0.83

## Abbreviations

EC: endometrial cancer
EIF4E-BP1: eukaryotic translation initiation factor 4E-binding protein 1
EMT: epithelial-to-mesenchymal transition
*SRA*: steroid receptor activator
siRNA: short interfering RNA
3'-UTR: 3'-untranslated regions
5'-UTR: 5'-untranslated regions
ncRNAs: Non-coding RNAs
mTOR: mammalian target of rapamycin
VEGF: vascular endothelial growth factor
