# Supplementary material for: Long non-coding RNA steroid receptor activator promotes the progression of endometrial cancer via Wnt/ β-catenin signaling pathway
Source: Int J Biol Sci. 2020 Jan 1;16(1):99–115. doi: 10.7150/ijbs.35643 (PMC6930375; doi:10.7150/ijbs.35643)
Supplement: Supplementary file 1 — Supplementary figures and tables. [file ijbsv16p0099s1.pdf]

**Supplementary Table 1. si*SRA* sequences used in the present study.**

| No | siRNA | | Duplex sequence | MW |
|---|---|---|---|---|
| 1 | *SRA1* | Sense | 5’ CUCAUAACAUGCAUUUCAAUU 3’ | 6227 |
| | | Antisense | 5’ UUGAAAUGCAUGUUAUGAGUU 3’ | 6364.1 |
| 2 | *SRA2* | Sense | 5’ GCUCAAGUGAGACACUUAAUU 3’ | 6345.1 |
| | | Antisense | 5’ UUAAGUGUCUCACUUGAGCUU 3’ | 6276 |
| 3 | *SRA3* | Sense | 5’ ACGUGAAUGUGGAGAUUGUUU 3’ | 6419.2.1 |
| | | Antisense | 5’ ACAAUCUCCACAUUCACGUUU 3’ | 6202 |
| 4 | *SRA4* | Sense | 5’ CAAGGAAGCAGGUAUGUGAUU 3’ | 6464.2 |
| | | Antisense | 5’ UCACAUACCUGCUUCCUUGUU 3’ | 6171.9 |
| 5 | *SRA5* | Sense | 5’ GUCCUAUUUGCACUGUAUCUU 3’ | 6212.9 |
| | | Antisense | 5’ GAUACAGUGCAAAUAGGACUU 3’ | 6408.2 |
| 6 | *SRA6* | Sense | 5’ CUGUAUCACCCUUGAGAUAUU 3’ | 6259 |
| | | Antisense | 5’ UAUCUCAAGGGUGAUACAGUU 3’ | 6362.1 |

**Supplementary Table 2. siEIF4E-BP1 sequences used in the present study.**

| No | siRNA | Duplex sequence | | MW |
|---|---|---|---|---|
| 1 | EIF4E-BP1_1 | Sense | 5’ GCAAUAGCCCAGAAGAUAAUU 3’ | 6391.2 |
| | | Antisense | 5’ UUAUCUUCUGGGCUAUUGCUU 3’ | 6229.9 |
| 2 | EIF4E-BP1_2 | Sense | 5’ GUUUGAGAUGGACAUUUAAUU 3’ | 6364.1 |
| | | Antisense | 5’ UUAAAUGUCCAUCUCAAACUU 3’ | 6227 |

**Supplementary Table 3. Primer sequences used in the present study.**

| Gene | Primer sequence | | Product size (bp) |
|---|---|---|---|
| | Forward (5’-3’) | Reverse (5’-3’) | |
| *SRA* | CTCCCTTCTTACCACCACCA | TGCAGATACACAGGGAGCAG | 217 |
| β-catenin, | TGCAGTTCGCCTTCACTATG | ACTAGTCGTGGAATGGCACC | 162 |
| Gsk-3β | AACTGCCCGACTAACACCAC | ATTGGTCTGTCCACGGTCTC | 154 |
| c-Myc | GGACGACGAGACCTTCATCAA | CCAGCTTCTCTCAGACGAGCTT | 92 |

[i] SRA, steroid receptor RNA activator.

A

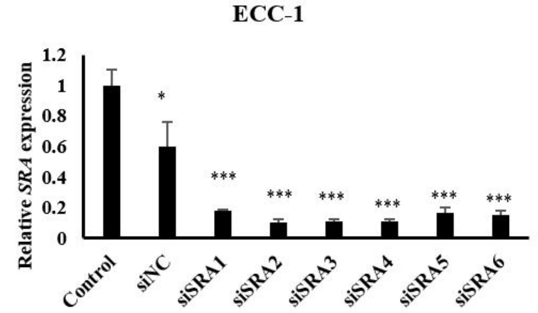
ECC-1
Relative SRA expression
1.2
1
0.8
0.6
0.4
0.2
0
*
***
Control
siNC
siSRA1
siSRA2
siSRA3
siSRA4
siSRA5
siSRA6

B

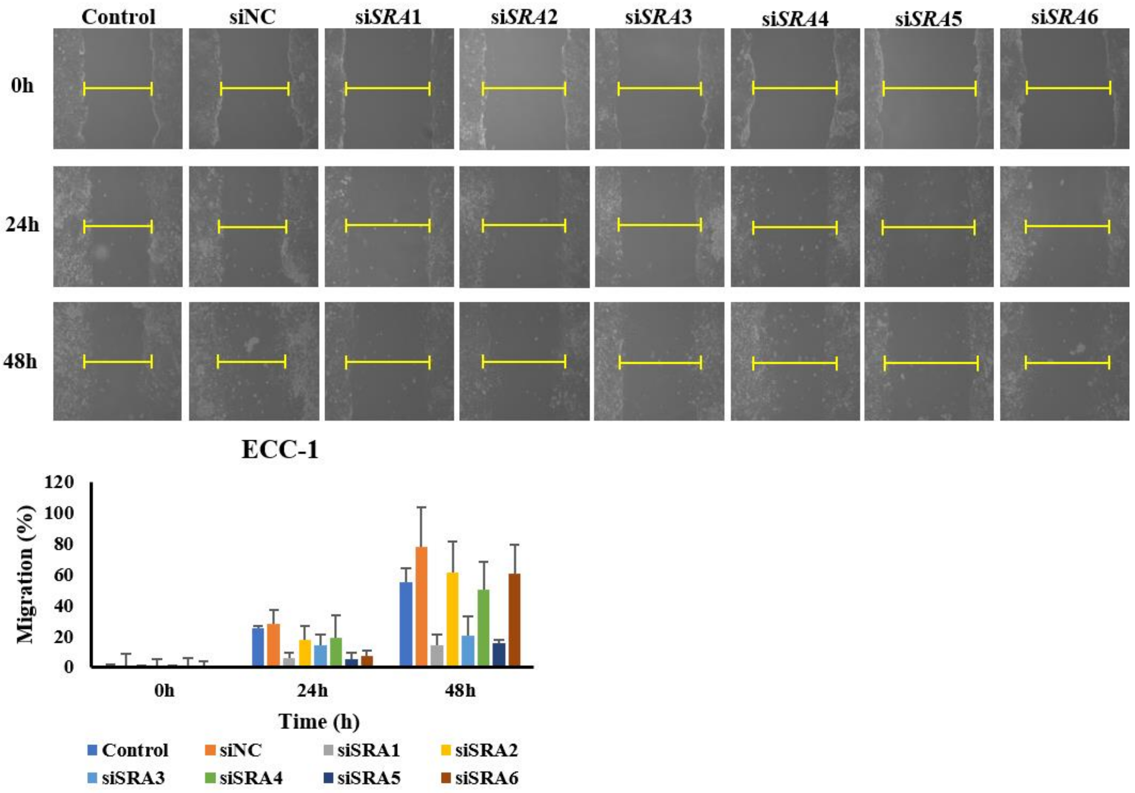
Control
siNC
siSRA1
siSRA2
siSRA3
siSRA4
siSRA5
siSRA6
0h
24h
48h
ECC-1
Migration (%)
120
100
80
60
40
20
0
0h
24h
48h
Time (h)
Control
siNC
siSRA1
siSRA2
siSRA3
siSRA4
siSRA5
siSRA6

C

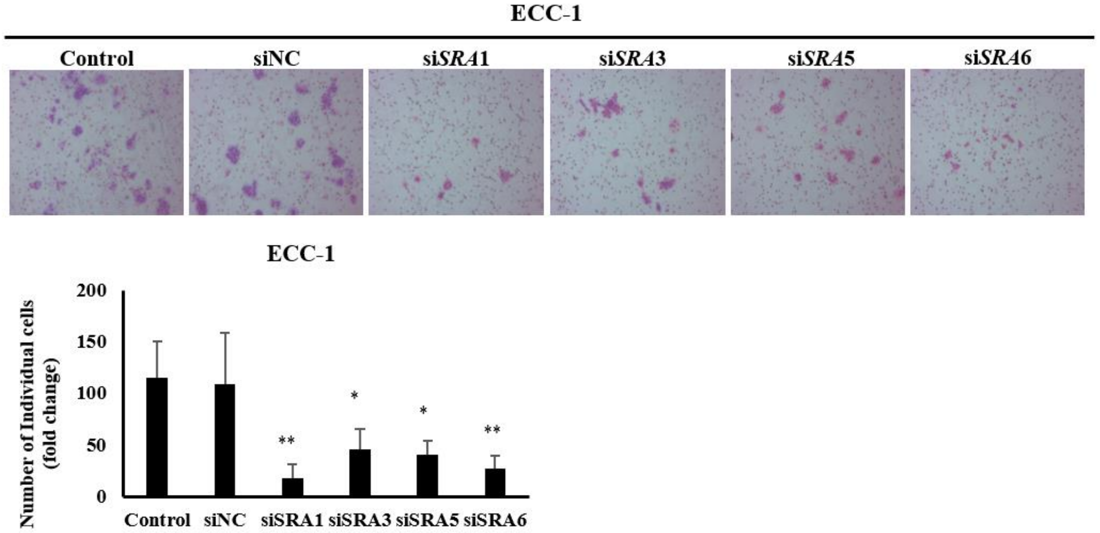
ECC-1
Control
siNC
siSRA1
siSRA3
siSRA5
siSRA6
ECC-1
Number of Individual cells (fold change)
200
150
100
50
0
**
*
*
**
Control
siNC
siSRA1
siSRA3
siSRA5
siSRA6

**Supplementary Figure. 1. Knockdown of *SRA* inhibits endometrial cancer *SRA* expression, migration, and invasion to minimize the possibility of off-target effects.** (A) Knockdown efficiency was determined by qRT-PCR analysis in ECC-1 cells. Cells were transfected with *SRA* siRNA (si*SRA*) or negative control siRNA (siNC and Control). (B) Wound healing assay was used to determine migration in *SRA*-specific siRNA (si*SRA*)-transfected ECC-1 cells (×200). (C) Matrigel invasion assay was used to determine invasion after 48 h in si*SRA* transfected ECC-1 cells. Bars indicate mean ± standard deviation of three independent experiments performed in triplicate. *$p < 0.05$, **$p < 0.01$, and ***$p < 0.001$ vs. siNC.

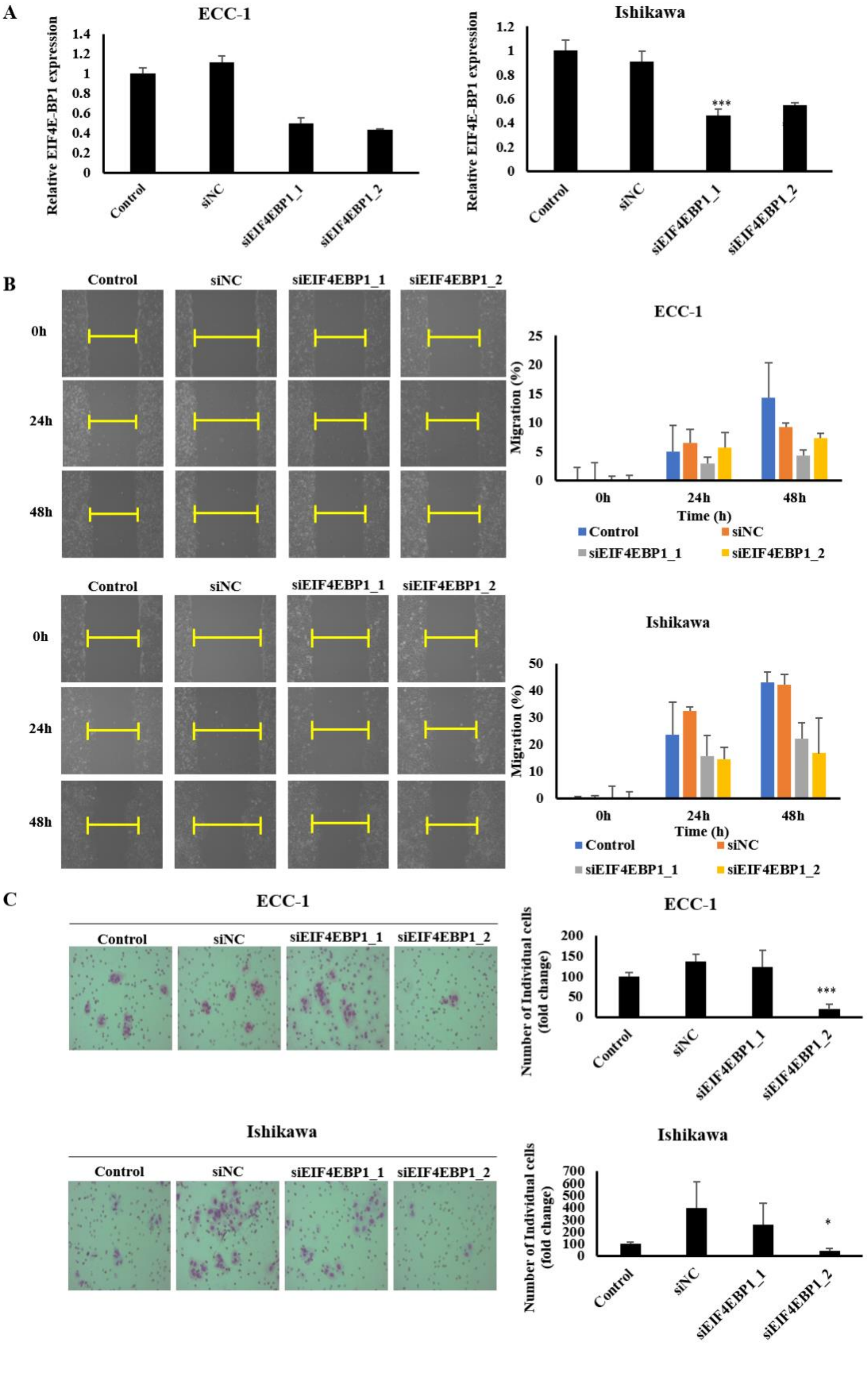

A
ECC-1
Relative EIF4E-BP1 expression
Control
siNC
siEIF4EBP1_1
siEIF4EBP1_2
Ishikawa
***
B
Control
siNC
siEIF4EBP1_1
siEIF4EBP1_2
0h
24h
48h
ECC-1
Migration (%)
Time (h)
Ishikawa
C
ECC-1
Number of Individual cells (fold change)
***
Ishikawa
*

**Supplementary Figure. 2. Knockdown of EIF4E-BP1 inhibits endometrial cancer EIF4E-BP1 expression, migration, and invasion to minimize the possibility of off-target effects.** (A) Knockdown efficiency was determined by qRT-PCR analysis in ECC-1 and Ishikawa cells. Cells were transfected with EIF4E-BP1 siRNA (siEIF4E-BP1) or negative control siRNA (siNC and Control). (B) Wound healing assay was used to determine migration in EIF4E-BP1-specific siRNA (siEIF4E-BP1)-transfected ECC-1 and Ishikawa cells (×200). (C) Matrigel invasion assay was used to determine invasion after 48 h in siEIF4E-BP1 transfected ECC-1 and Ishikawa cells. Bars indicate mean ± standard deviation of three independent experiments performed in triplicate. *$p < 0.05$, **$p < 0.01$, and ***$p < 0.001$ vs. siNC and Control.
